# The History and Characteristics of the Mobulid Ray Fishery in the Bohol Sea, Philippines

**DOI:** 10.1371/journal.pone.0161444

**Published:** 2016-08-30

**Authors:** Jo Marie V. Acebes, Malcolm Tull

**Affiliations:** 1 Department of Biology, School of Science and Engineering, Ateneo de Manila University, Loyola Heights, Quezon City, Philippines; 2 BALYENA.ORG, Paseo del Mar, *Barangay* Pangdan, Jagna, Bohol, Philippines; 3 School of Management and Governance, Murdoch University, Perth, Western Australia; Leibniz Center for Tropical Marine Ecology, GERMANY

## Abstract

The fishery for mobulid rays, also known as devil rays, has been practiced in the Bohol Sea for over a century yet very little is known about its history and characteristics. This study provides the first detailed description of the mobulid ray fishery in the Bohol Sea, Philippines. It describes the history and evolution of the fishery from the 19^th^ century to 2013. It characterizes the fishery based on the species targeted, gears used, the organization, catch distribution, processing, monetary value, and the market of its by-products. This paper also analyses the changes that occurred through time, the management of the fishery and the drivers of the fishery. A multi-disciplinary approach was employed by combining ethno-historical research methods and catch landing monitoring in four primary sites within the Bohol Sea. This fishery began as an artisanal fishery using sail and row boats equipped with harpoons and gaff hooks practiced in at least four coastal villages in Bohol, Camiguin and Limasawa. The fishing fleet has decreased since the beginning of the 20^th^ century however, with the motorization of the fishery and shift to the use of gillnets, the extent of the fishing grounds and market of the products have expanded. Four species of mobulid rays are caught in the Bohol Sea: *Manta birostris*, *Mobula japanica*, *Mobula thurstoni* and *Mobula tarapacana*. A fifth species, targeted by a fishing community off Dinagat as an off-shoot of the Bohol fishery is most likely the *Manta alfredi*. Currently, the fishery for mobulids is centered in Bohol Province where it has been practiced longest. The monetary value of mobulids in this region has increased and the dependence of fishing communities for their livelihood is significant. The unique characteristics of this fishery and the socio-cultural context within which it operates merits a thorough investigation in order to design the appropriate management strategy.

## Introduction

Devil rays fall under the family Mobulidae, which is represented by the two genera *Manta* and *Mobula* [1], and are generally referred to as mobulid rays or mobulids. Mobulids have been documented since at least the 17^th^ century yet little information is available on their ecology and biology [1]. Worldwide, there are at least 11 species of mobulids, seven of which are recognized to occur in the Philippines, *Manta birostris*, *Manta alfredi*, *Mobula tarapacana*, *Mobula japanica*, *Mobula thurstoni*, *Mobula kuhlii*, and *Mobula eregoodootenkee* [2–5]. Mobulids are often referred to as devil rays because of the prominent cephalic lobes or fins located on the front of their heads giving them the appearance of having “horns” [6]. The body or disc of all species of mobulids is lozenge or diamond-shaped. Two species of manta rays are recognized: the giant manta ray (*Manta birostris*) and the reef manta ray (*Manta alfredi*) [7]. The two species appear very similar and in this study, the term manta ray is used to refer to the giant manta ray, unless otherwise specified. Both species are distributed worldwide [7]. Known as manta, or more recently oceanic manta ray or pelagic manta ray, the giant manta ray is the largest of the family Mobulidae with a disc-width that can reach over 7 meters [1, 5, 7, 8, 9]. With a very broad head and mouth that is terminally located, it has no teeth in the upper jaw [5, 7, 9]. It has long, prominent cephalic lobes and the tail is shorter than the disc [9]. The body surface is rough and the colour of the dorsal surface or back is black or greyish blue to greenish brown, often with irregular pale shoulder patches [9]. The ventral surface is white with irregular and elaborate dark patches or spot patterns which can be used to photo-identify individual animals [7]. Giant mantas feed on small planktonic organisms, like other devilfishes [9]. Little is known about the species’ reproductive biology, but reports indicate it gives birth to a single pup per litter [7]. The giant manta ray can be found in shallow waters near reefs to open oceans [5]. In the Philippines, little is known about their distribution. Aside from records of catches in the Bohol Sea, sightings of divers in the Visayan Sea (off Malapascua Island, northern Cebu), Ticao Pass and Burias Pass off the coasts of Masbate Island, nothing is known about the extent of their distribution [10].

There are nine recognized species under the genus *Mobula* which range from one to five meters in disc width [1]. A manta ray can be differentiated from a mobula ray based on several physical characteristics, namely the size, position of the mouth, the texture and color of the skin and the length of the tail. The mouth of the mobula is subterminal and faces downward, unlike the mouth of the manta ray which is terminal and faces forward [2]. Five out of nine mobula species are known to occur in the Philippines [2]: the spinetail mobula (*Mobula japanica*), also known as the Japanese devil ray; the bentfin devil ray (*Mobula thurstoni*), also known as the smoothtail mobula, lesser devil ray, or Thurston’s devil ray; the sicklefin devil ray (*Mobula tarapacana*), also known as the Chilean devil ray or spiny mobula [2, 5]; the shortfin devil ray (*Mobula kuhlii*); and the longhorned mobula (*Mobula eregoodootenkee*) or the pygmy devil ray. Mobulids have a circumglobal distribution and are found in tropical and temperate off-shore and continental waters [7–9]. The exact distribution of all three *Mobula* species in the Philippines is not known.

All species of mobulids are known to be directly or incidentally caught [1, 11, 12]. The fishery for mobulid rays has received much attention recently because of the worldwide concern for decreasing populations and the increase in demand for its by-products [1,6,7, 13]. Mobulids, similar to other large marine vertebrates (i.e. whales, sharks), have long life histories, slow growth and reproductive rates which make these animals susceptible to exploitation [13–16].

The fisheries for mobulids are poorly documented [12]. There are only a few places in the world where these fisheries are described in any detail. Some mobulids are caught as target species while others are products of by-catch. In the Gulf of California they are targeted using harpoons and gill nets while in New Zealand, and the tropical Western and Central Pacific purse seine tuna fisheries, mobulids are only by-catch [12]. In the Indonesian artisanal gillnet fishery, mobulid rays are also taken as by-catch [12]. In several sites in southern Indonesia, a large proportion of rays are caught as by-catch from tuna gillnet, longline fisheries, drift gillnet, and trammel nets [17]. In Eastern Indonesia, fishers of whales and whale sharks also take rays, using handheld harpoons [18,12]. According to Dewar [19], in Lamakera (Indonesia) the fishery has recently shifted to targeting manta rays. Directed fisheries also occur in Sri Lanka, India and Thailand [6, 8, 12]. In a report by the Manta Trust [20] on Sri Lanka’s manta and mobula fishery, it is stated that rays are mostly caught as by-catch in gill nets.

In the Philippines, rays and sharks have been hunted for centuries. Seventeenth century accounts by missionaries of Filipinos in the Visayas describe how people caught, ate and utilized shark and ray products [21]. The Philippines’ trade with the Chinese in marine resources from Sulu such as tortoise shells, shark fins and rays has been documented in the 18^th^ and 19^th^ centuries [22, 23, 24]. Harpooning of rays appears to have been practiced across the Visayas and Sulu archipelago [21, 25]. Sharks and rays were considered as some of the commercially important marine resources of the Philippines in the early 1900s and were abundant yet their fisheries were deemed unexplored or underutilized until the late 20^th^ century. Fishers in the Bohol Sea have hunted rays since at least the late 19^th^ century using harpoons and gaff hooks. Rays then were found in great abundance along the coasts. The development of fishing technology, changes in the economy, improvement of the transport system and expansion of the market all contributed to the increase in fishing pressure for rays in the Bohol Sea. Six species of mobulid rays, including the giant manta ray (*M*. *birostris*), are caught either incidentally or directly throughout the country. Although *M*. *birostris* has been protected in the Philippines since 1998, to this day it continues to be caught.

The primary objective of this paper is to provide the first detailed description of the mobulid ray fishery in the Bohol Sea, Philippines. It traces the history of the mobulid ray fishery in the Bohol Sea from the 19^th^ century to 2013 and describes the evolution of the fishery and its current state. Secondly, the paper documents the characteristics of the fishery by providing details of the implements used, the organization, catch distribution, processing, monetary value, and the market. This paper also describes the target species of the fishery, the fishing grounds and the main fishing seasons. Finally, it provides an analysis of the changes that occurred through time, the management of the fishery and the drivers of the fishery.

## Methods

The Murdoch University Human Research Ethics Committee approved the Project No. 2010/011 entitled: “Historical catches of large marine vertebrates in the Bohol Sea: interactions of communities with their marine environment, socio-economic changes and conservation management implications in the Philippines” on 2 February 2010. This PhD research project was largely the basis of this study. Prior to the conduct of the interviews, a project information letter was presented to the mayor of each municipality where the interviews were going to take place. Permission was granted to the investigator to conduct the interviews in each of the sites. Participants in the study gave either verbal or written consent. Those who gave written consent signed the consent form prepared before the start of the interview. Other respondents who were not comfortable signing their name agreed to participate in the study by giving their consent verbally. Most of the respondents were fishers above the age of 60 whose highest educational attainment was in the primary level (elementary) or secondary level (high school). In view of the background of the respondents, the ethics committee approved this consent procedure.

**Fig 1:**
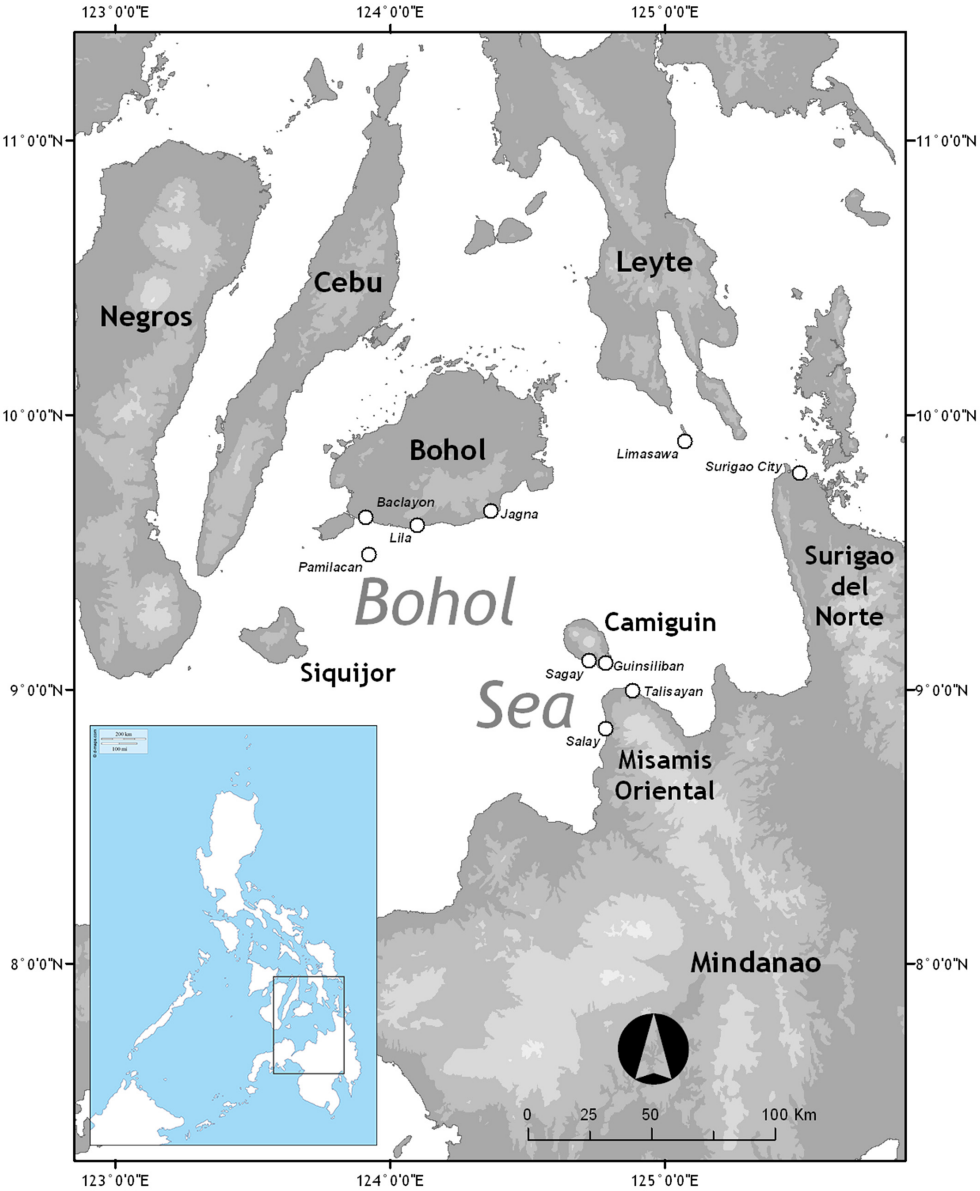
Map showing study sites: Jagna, Lila, Pamilacan, Camiguin and Limasawa.

This study covers the Bohol Sea, also known as the Mindanao Sea, in the central part of the Philippines. It is located between the Visayas and the greater island of Mindanao at approximately 9°N 124°E. It covers 29,000 sq km of waters fronting the southern part of Bohol Island, western Surigao del Norte, northern Mindanao and eastern Siquijor [26] (Fig 1). There were five main field work sites: Jagna, Lila, Pamilacan in southern Bohol; Sagay in Camiguin; and Limasawa in Southern Leyte. An additional site in a coastal village off Dinagat Province was also visited in 2010.

The multi-disciplinary approach of marine environmental history, especially that of the History of Marine Animal Populations project [27], was utilized in this study by combining data obtained from ethno-historical research with biological data from published and unpublished sources (i.e. unpublished scientific reports) [28,29]. Due to limited historical source material available in archives and libraries, the history of these fishing practices was reconstructed by combining oral history, fragmented archival and government records, published travel narratives, missionaries’ accounts, several non-government organization (NGO) reports, web and print news articles. Additional data were gathered from participant observation, recent fisheries data and actual catch landings monitoring.

Archival research was conducted in the Philippines, Spain and the United States between November 2010 and September 2011. In the Philippines, research was conducted in the Philippine National Archives, the Philippine National Library, Filipinas Heritage Library, *Archivo Recolletos*, Silliman University Marine Lab Library, the University of San Carlos libraries, municipal and provincial libraries in Bohol. In Spain, archival research was conducted at the *Biblioteca Nacional* and *Archivo de Indias* while in the USA, at the National Archives II at College Park.

At the field sites, semi-structured key informant interviews were conducted with municipal fisheries officers, *barangay* (village) captains, boat owners, buyers, fishers, former hunters and current fishers, processors and other community members involved in the fisheries. NGO staff members working in the Bohol Province, members of the academe in the province and members of community cooperatives were also interviewed. Bureau of Fisheries and Aquatic Resources (BFAR) officials and Manila-based NGO officials and former staff were also interviewed.

Through document or contents analysis, the history and evolution of the fishery were constructed. This involved qualitative analysis of relevant documents in order to create a timeline of events from different perspectives and coding data into different themes or categories [30]. This was combined with data from the qualitative interviews. Information gathered from anecdotes and memories of old fishermen and other key informants, travel narratives, naturalists’ journals, and other old documents were used to construct the history of the Bohol Sea and its exploited species [31].

Participant observation and catch landing monitoring was conducted in the fishing village of Bunga Mar in Jagna, Bohol between the periods of April to August 2010, October to November 2010, January to February 2011 and April to June 2011. Pamilacan Island was also visited on June 2010, May and June 2011 and observations and interviews were conducted for several days at each visit. Three fishing villages in Sagay, Guinsiliban and Catarman on Camiguin Island and two fishing villages in Limasawa Island were visited in October and November 2010, respectively. A fishing village off Dinagat was visited in June 2010 where interviews were conducted.

The target species of the fishery were determined by actual observation of catches landed in study sites with active fisheries. Photographs were taken of the catches landed for further verification by comparison with published ray identification guides. Data on fishing effort were obtained from actual observation and recording of the number of boats that go out and land catches in the fishing village, and counting and identification of species landed per boat per day for at least three consecutive days during the site visits. The fishing grounds were determined by asking fishers to mark on a map the location where they drop their nets. In order to get a more accurate record of the location of their fishing grounds and travel route, a handheld Global Positioning System (GPS) was given to a fisher once in 2010 and again in 2011.

## Results and Discussion

### Target species

Rays are generically called *page* or *pagi* in the Philippines. This has created confusion among fisheries researchers and managers because there are several species and some are quite difficult to distinguish from one another, except to those who catch them. In Bohol the manta ray is referred to as “*sanga*” while all the other mobulas are generally lumped into one group called “*pantihan*”. However, some expert fishers and buyers in Bohol distinguish three other types of mobulids, the “*salindangan*” (in Jagna) or “*pilong*” (in Pamilacan), which they differentiate from other rays based on colour and texture of the skin; and the “*binsulan*” and the “*masinaw*”, which they differentiate based on the presence or absence of a spine. Based on examination of several specimens in landing sites during this study, it can be said that what the Boholanos refer to as “*sanga*” refers to the giant manta ray (*Manta birostris*), “*salindangan*” or “*pilong*” is the sicklefin devil ray (*Mobula tarapacana*), while the “*binsulan*” is the spinetail mobula (*Mobula japanica*) and the “*masinaw*” is the bentfin devilray (*Mobula thurstoni*). From inference, since fishers from Bohol and Camiguin utilized the same fishing grounds, the manta rays hunted by fishers from Camiguin were also the giant manta ray.

In Leyte and Surigao, manta rays are called “*saranga*”. Actual specimens were not examined during this study hence it was difficult to determine whether these were in fact giant manta rays or the reef manta ray (*Manta alfredi*), or a different species of *Mobula*. However, based on descriptions of fishers from Dinagat, the species targeted in the Surigao Strait are different from the manta rays hunted in the Bohol Sea. It is highly likely that it is the *Manta alfredi*.

A study conducted in 2002 and 2010 by Rayos *et al*. [32] noted the landing of *Mobula kuhlii* and *Mobula eregoodootenkee* in Jagna, Bohol. However, these two species were not observed to be landed during this study. None of the respondents described seeing these two species or any other species apart from the five species mentioned above.

### History and characteristics of the fishery

Based on archival records, the fishery for mobulids in the Philippines and elasmobranchs in general can be traced back to the 17^th^ century. In the Bohol Sea, according to oral history, the practice of hunting manta rays goes back as far as the late 19^th^ century, further in time than the practice of whaling [10]. Fishers from the villages in Lila, Pamilacan and Jagna in Bohol, those from Sagay and Guinsiliban in Camiguin and Magallanes in Limasawa hunted manta rays. The fishers from Lila and Pamilacan and Camiguin also hunted whales [10]. Mobulid ray hunting in Jagna, Bohol appears to have been practiced longer than in any other town around the Bohol Sea.

The history of the mobulid fishery can be divided into two periods: the earlier period from 1800s to the 1960s and the mechanized period from the 1970s onwards. The characteristics of the fishery in each period are discussed in the following order: technology of the fishery, distribution and utilization of the catch and fishing effort and organization.

#### The fishery from the 1800s to 1960s

**Fig 2:**
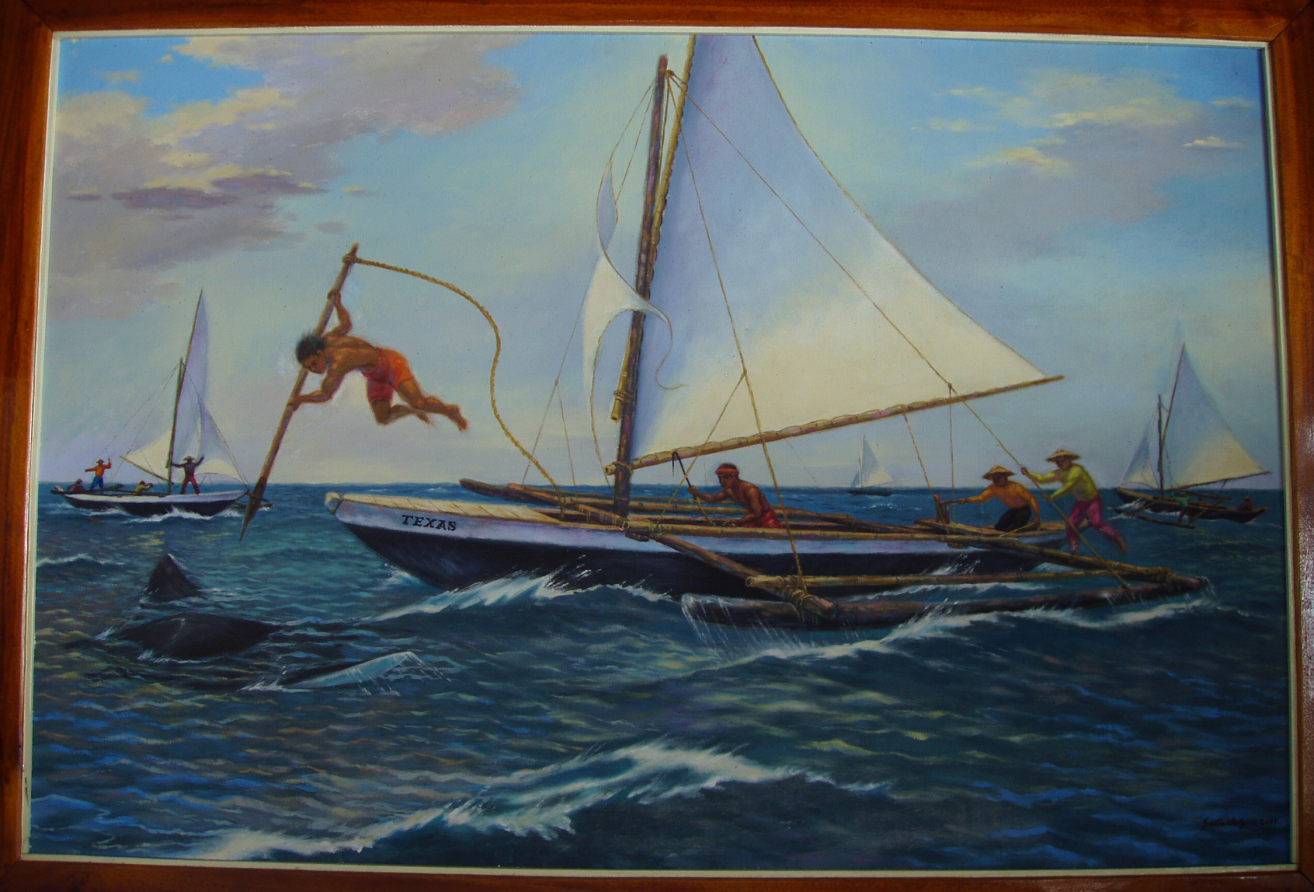
A painting depicting the technique of hunting for manta rays in Jagna, Bohol by Salio Ortiz, 2007

The technique of hunting manta rays is similar among all main study sites in Bohol, Camiguin and Limasawa. The manta is caught by a fisher jumping on top of the animal and thrusting a sharp fishing implement, a hook or a harpoon, from a wooden outrigger boat (Fig 2). The size of the boat, crew and the type of fishing implement used varied among sites. Fishers used wooden outrigger sail and row boats ranging from nine to ten meters long at Lila and Sagay; eight to 14 meters at Jagna and 5.5 meters at Limasawa. Those with sails were woven from dried plant leaves local to the island or flour sack cloths.

**Fig 3:**
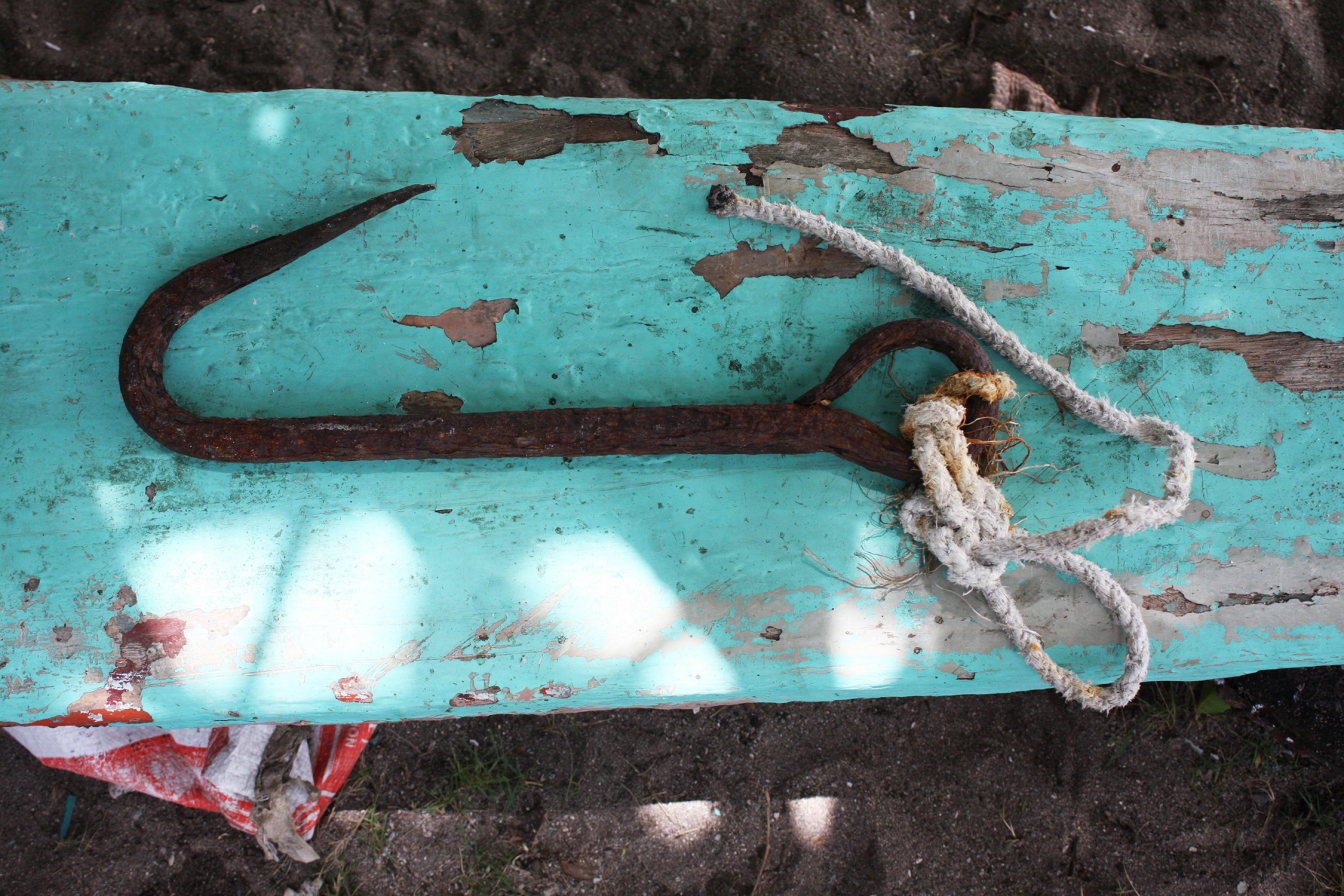
Hook or *pilak* used by Pamilacan whale and manta ray hunters.

**Fig 4:**
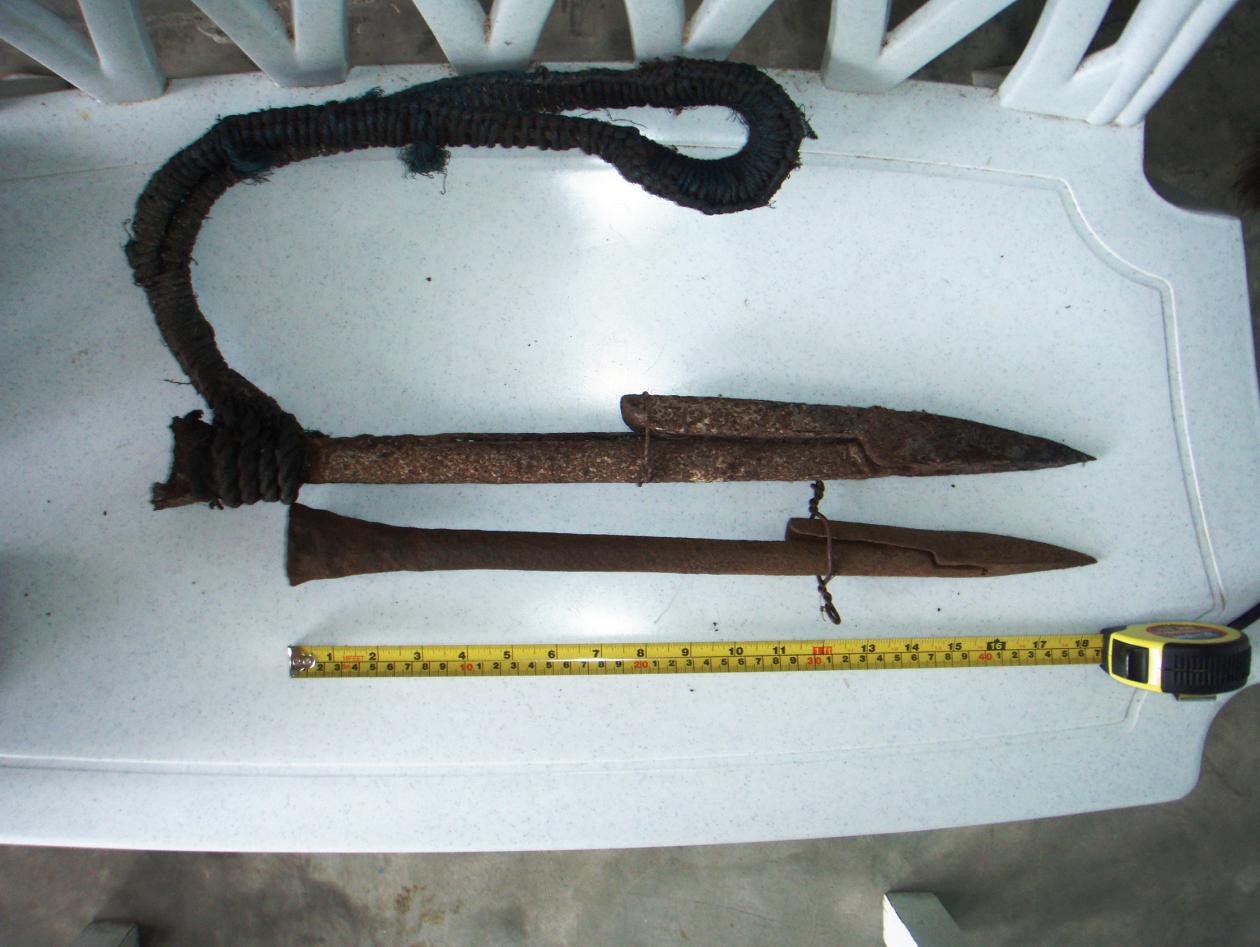
Harpoon or *isi* used by fishers from Jagna (top) and Camiguin (bottom) to catch manta rays.

In Lila and Pamilacan, Bohol, a large iron hook called “*pilak*” about 47cms long was used (Fig 3). In Limasawa, fishers used an iron hook called “*taga*” about 60 cm long, similar to that used in Bohol. In Jagna, a locally crafted toggle harpoon called “*isi*” similar to that used by American 19th century whalers was used (Fig 4). The harpoon tip was attached to the end of a 3-metre long wooden pole and secured with a rope. Fishers also used a “*gangso*” or gaff hook to secure a harpooned manta ray and a long knife or “*sundang*” to later kill it and cut it up. In Sagay, Camiguin fishers used a harpoon with the exact likeness of that used in Jagna.

The number of crew members varied slightly among the communities but the main duties or roles were essentially the same. The boats were manned by a crew of at least two (in Limasawa) to a maximum of six men (in Lila and Pamilacan). In Jagna, they had three to four men. There are three main roles on the boat: the oarsmen or rowers, the helmsman who usually alternates as an oarsman, and the most important member of the crew, the “jumper” or “hookman”. There may or may not be one or two paddlers who were usually young apprentices. These roles however, were not fixed in the sense that although each crew member had a main role they also had other responsibilities during the hunt and also helped each other in performing those responsibilities. The “jumper” was likened to the captain of the boat. He was responsible for his crew and directed them what to do. He directed where the boat should go when in pursuit of a ray.

Fishing was done during the day, leaving in the early morning and returning as soon as there is a catch. In Lila, Pamilacan, Sagay and Limasawa, the “jumper” was perched at the bow on the lookout for mantas while three or four were oarsmen, with one acting as the helmsman controlling the steering oar. Using his one hand holding the hook, the “jumper” directed the helmsman, pointing it to which direction the animal was moving. Once he (and the bow of the boat) was about arms-length from or almost directly above the animal, he jumped thrusting the hook into the manta ray’s back. The rope attached to the hook, which was securely tied to the centre post of the boat, was guided by one of the oarsmen. The rope was about 120 m long and was paid out gradually, a few meters at a time, until it was almost taut. They allowed the manta to drag the boat until it became tired. Depending on the size of the animal, another boat may try to help by attaching another hook unto the ray. Once subdued, the “jumper” went back in the water to stab and kill it. The manta ray was then tied up around its cephalic lobes and around the base of the tail and secured to the side of the hull of the boat; it was then dragged to shore.

All communities mentioned hunted manta rays using the same fishing implements and techniques they used for catching whales. The only variation was that fishers did not use floaters for manta rays. The rope attached to the hook or harpoon was directly tied to the boat. According to respondents, manta rays were easier to catch than whales without assistance from other boats.

In Jagna, fishers in the village of Bunga Mar specialized in hunting only manta rays. The hunting technique varied only slightly from what was earlier described. Unlike in other communities previously described however, in Jagna, the manta ray was towed back to shore alive.

The hunting season for manta rays coincided with the season for whaling since the animals went after the same prey, the “*uyabang*” (krill). It would start from the month of March until May. There were seasons however, when fishers started the hunt on January or even as early as December when the manta rays “appeared” earlier. Fishers believed that they could catch the manta rays whenever krill were present because that was when they would go to the surface to feed. Although the arrival of the rays coincided with the “calm season”, from the months of February until June, there was no guarantee of a catch.

Fishers in the past need not go far to hunt for manta rays. The beginning of the season was signaled by the sight of mantas leaping from the water which people clearly saw from shore. The common fishing grounds were all along the coasts of southern Bohol, northwestern to southern coasts of Camiguin and eastern coasts of Limasawa, extending no further than five kilometres from shore.

The primary product of the fishery is the mobulid meat. However, the skin, parts of the gill rakers and most of internal organs were also utilized. In Bohol and Limasawa, the manta caught was divided equally among each member of the crew and the boat owner. However, the jumper, aside from his share, received a prized part of the manta, the “*paa-paa*”. If more than one boat participated in the hunt, the catch was divided into five: four shares went to the boat that hooked the ray and one share went to the boat or boats that helped. Each fisher was free to do as he wished with his share.

Manta meat cut into square pieces was sold fresh or dried with skin intact. Most often the skin was just thrown away. There was no distinction between dark or white meat, the price was the same. The gill rakers were eaten too but not in their entirety, as only a portion is edible. Lila fishers sold manta meat to other nearby towns while Pamilacan fishers sold their catch whole to Lila, Baclayon and Panglao.

The system of distribution of the manta ray catch at Sagay and Catarman, Camiguin differed very little from that in Bohol. Half of the catch was given to the boat that harpooned the manta while the other half went to the boats that helped. Only a few boats cooperated in the hunt hence the half that goes to the boats that helped was usually divided only into two or three. Each half was then divided equally among the crew of the boat including the owner of the boat and the harpoon (if different). According to some respondents, sometimes the harpooner was given an additional share as a prize and the preferred part was the tip of the “wing” of the manta while the oarsmen received the cephalic lobes. Manta meat was sold locally within the Island. Meat that was not sold fresh was dried.

**Fig 5:**
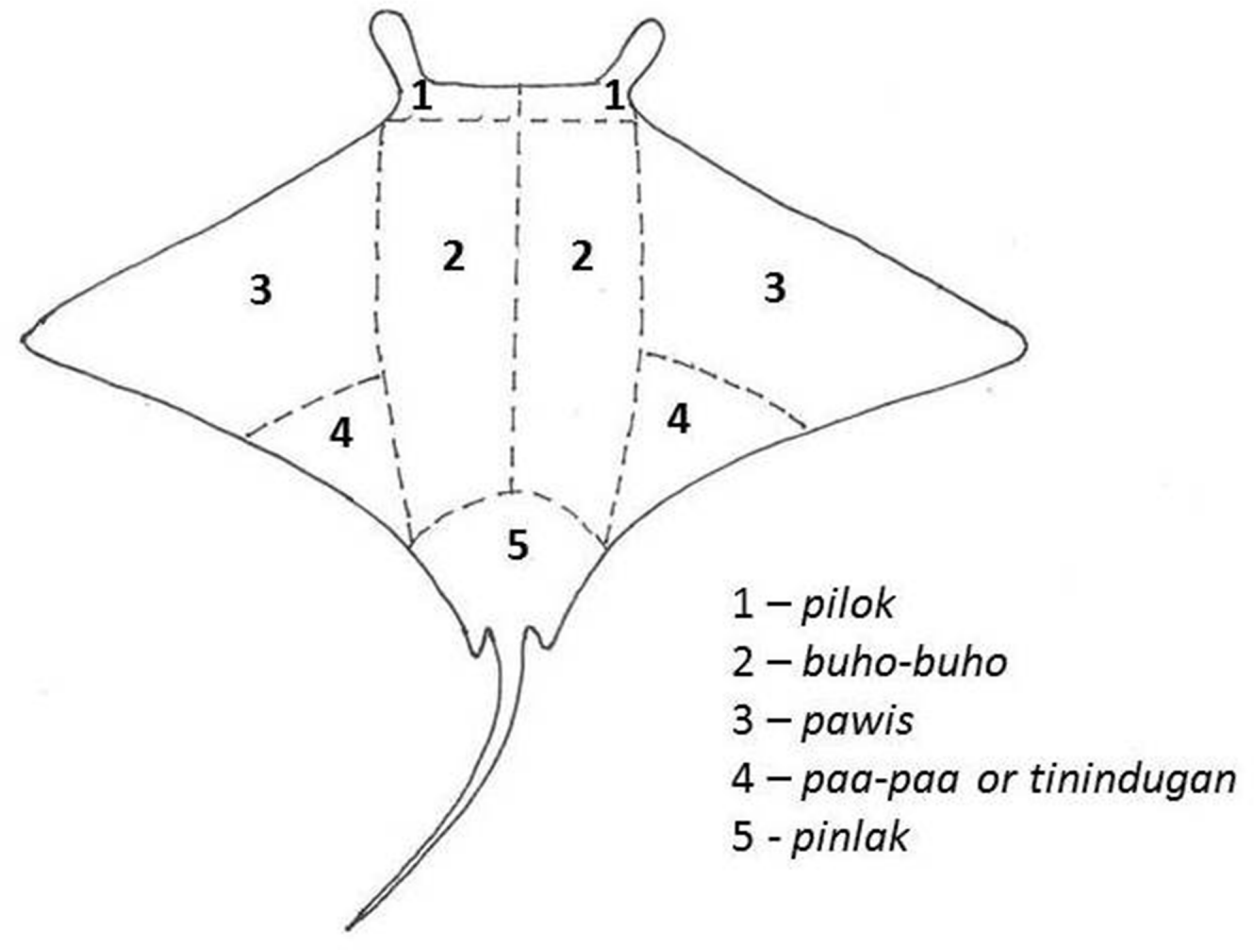
Illustration of the division of the manta ray as described by respondents.

The system of distribution of the catch in Jagna was described in more detail by Yap [33]. According to Yap [33], the manta ray was divided into six primary parts but the method of dividing varied according to how the ray was caught and shares were distributed according to the role the person played in the hunt. In no other fishing community was the system of distribution of the catch described in so much detail as that by Yap [33] for Jagna. Most aspects of his findings were corroborated by testimonies of elder respondents during this research (Fig 5). The share of the catch of each boat that participated in the hunt was divided into five: two shares to the boat owner (who usually owned the harpoon) and one share to each member of the crew. As in other fisheries described previously, each member of the crew was responsible for selling or bartering their share of the catch. Most of the meat and by-products were consumed by the family of the fishers and any excess was sold or dried and bartered to neighbouring villages and municipalities. The women were primarily responsible for taking the meat to the weekly market day called “*tabuan*” where people from different villages gather to sell their produce. Some took their products to other municipalities further inland by public land transport.

Prior to mechanization, a boat usually caught only one manta ray a day. On a good day, the entire fishing village caught five to ten mantas. At times when there was plenty of catch for several days, fishers had difficulty selling the meat. It was then that they dried any unsold meat and bartered it for corn and root crops.

According to Yap [33], during the first quarter of the twentieth century in Jagna there were around 300 sail boats that hunted for manta rays. Several elder interview respondents from Jagna estimated that in the 1950s, there were an estimated 30 to 50 manta ray fishing boats in operation.

Respondents attested that in the past, it was common to catch a manta ray about four to seven meters, and even up to nine meters wide. However, the fishery was still uncertain as there were days during the fishing season when “the manta ray did not let itself be taken”. For this reason, most fishers turned to so-called “experts” to perform a ceremony or ritual to “call the fishes” and the “sea spirits”. Fishers then believed in the practice of “*buhat-buhat*” wherein a known man with such expertise of being able to perform such a ceremony was called upon. The ceremony was conducted just before the start of the fishing season. Several respondents believed that performing the ceremony brought them a good catch and failing to do so brought not only no catch but also bad luck. It was said that the ceremony rendered the large fishes tame, allowing the fishers to catch them. As such the ceremony was performed not just for manta rays but for whales and whale sharks as well.

At a village in Limasawa Island, fishers hunted manta rays only until the middle of the 1980s. According to older respondents, there used to be from ten to 20 boats in the village that hunted manta rays. The catch was unpredictable and by the 1950s catching five mantas during the entire season was already considered lucky. The usual size of the manta ray caught was about 5.5 meters wide, but sometimes rays up to 7.3 meters wide were caught. Before the 1970s, Manta ray meat was not sold by the kilo but in piles estimated to be five kilos per pile.

The organization of the fishery was fluid with the crew members informally recruited and not being permanently tied to a particular boat or group of fishers. Although most crew members were related to each other, this was not sought intentionally but instead was mainly because village members were most often related by blood or marriage ties. The boat owner was also a fisher and was most often the “jumper”. There were no organized groups of labourers or processors. There were no separate groups of wholesale buyers and sellers. By the 1940s in Jagna, there were buyers to whom fishers would sell their catch to. The buyers processed the entire ray by themselves (with the help of family members) and sold them outside the towns.

The technique of using harpoons to catch manta rays is not unique to the Philippines. The Bajau Laut in Sabah hunted dolphins, porpoises and giant manta rays using harpoons [25]. The fishers of Lamalera in Nusa Tenggara Timur, on the island of Lembata in eastern Indonesia, hunted whales, dolphins, manta rays and sharks using the same technique of jumping onto the back of the animal and thrusting a locally crafted harpoon [18]. There are also striking differences, from the design of the fishing implements and the boat used, the organization of the fishery, the system of distribution, to rituals associated with the hunt. In Lamalera, the entire manta ray was taken into the boat where it was cut up into several sections and stored until the end of the hunt for the day. The manta ray was divided into “share locations” which were distributed accordingly to the members of the “corporation” [18]. Unlike in the Bohol Sea fishing communities where boats were owned by individuals and their immediate families, large fishing boats in Lamalera were owned and maintained by “corporations” [18]. Each boat was associated with a “great house” which was usually the centre of a clan or of one its segments [18]. Members of the corporation were not necessarily crew members, but normally the core crew were the most active members of the corporation. The corporation was headed by the “boat master” who was essentially the manager. The boat master’s immediate family and other people involved in the construction of the boat and the fishing implements were also part of the corporation. The system of distribution of the catch was intricate and varied from one corporation to another. Unlike in the Bohol Sea mobulid fishery, the crew could get additional shares if they spotted the ray first and if more than one ray was caught in a day. Furthermore, the corporation’s share in the manta ray was first dried before distribution.

Although ceremonies were also performed by fishing communities in the Bohol Sea, these were not as elaborate as in Lamalera. Fishers of Lamalera adhere to rituals and related systems of beliefs before, during and after the hunt. There are many prohibitions in speech and behavior, as well as specific songs for rowing and hunting [18]. Ceremonies were also performed in association with the different stages of boat building.

#### The fishery from the 1970s to 2013

**Fig 6:**
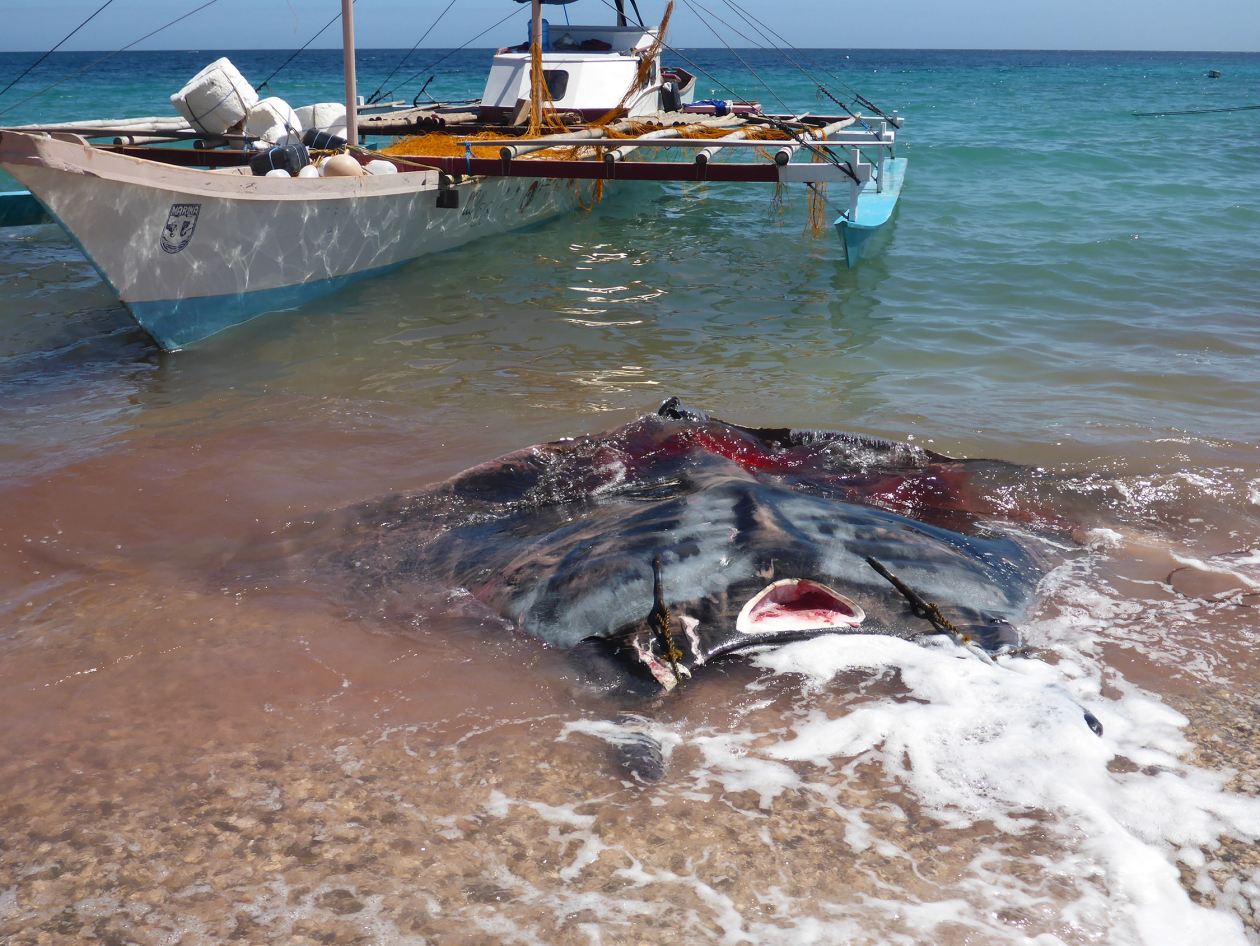
Canter boat with a landed giant manta ray in Jagna, Bohol.

The mechanization of the fishery began in the 1960s. At the onset, the use of harpoons and hooks were maintained until about the 1970s when synthetic (nylon) nets were introduced. With mechanization, the size and design of the boats also changed. Larger and more powerful engines became available and bigger boats were built. At first “pumpboats”, outrigger canoes powered by a small gasoline or diesel engine such as Briggs & Stratton engines, were used; then gradually larger boats called “canter” were built (Fig 6).

In 1997, for Pamilacan, the boats ranged from 8–16 m. By 2011, boats in Pamilacan ranged from 12-18m with Mitsubishi 6D14 or Fuso Canter 4DR5 engines. Currently, in Jagna boats are about 9-12m in length with 4D30 Mitsubishi, or 4DR5 Mitsubishi canter or 6D14 engines.

**Fig 7:**
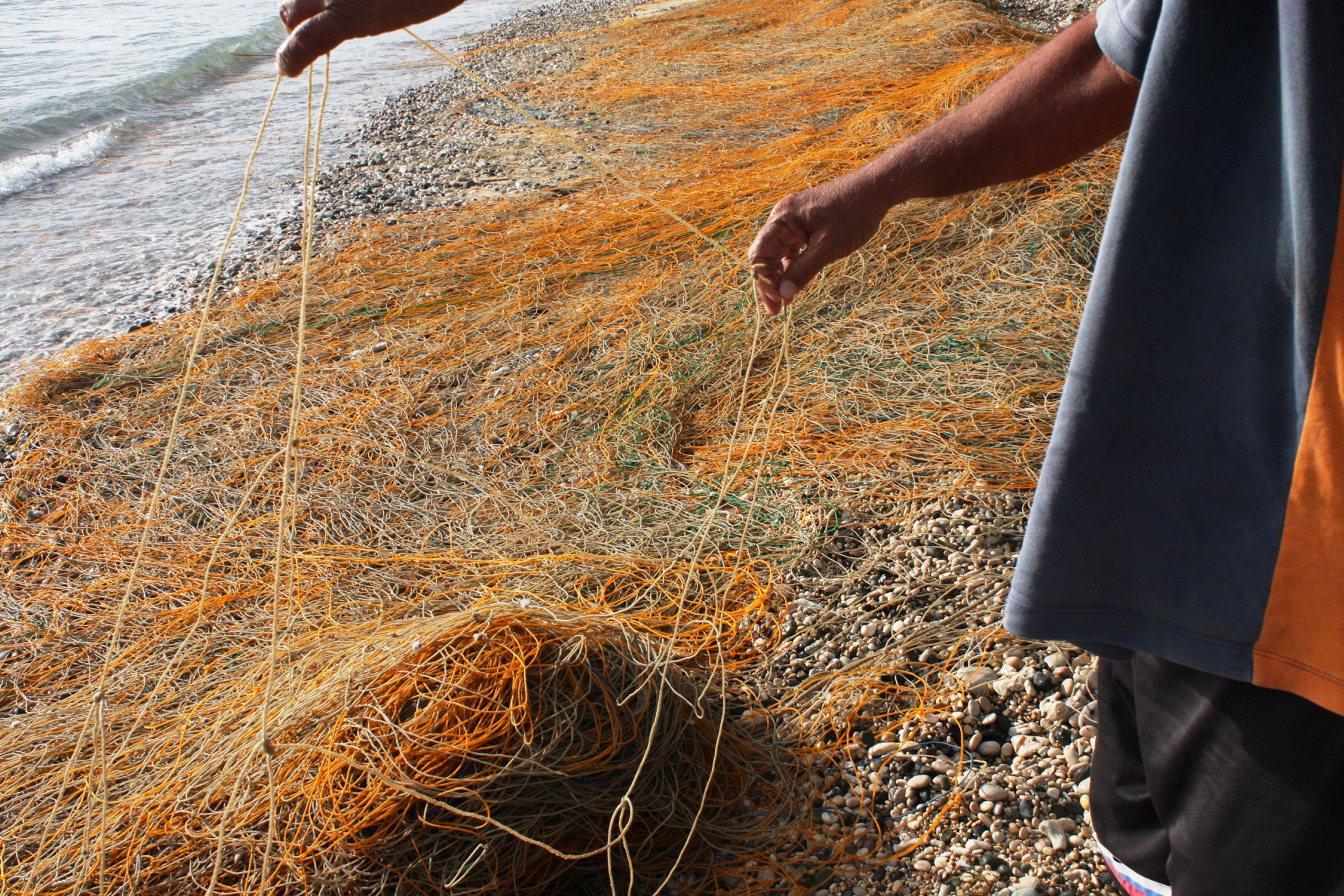
*Koralon*, net used for fishing mobulid rays.

In 1978 in Jagna, fishers started using synthetic nets, commonly referred to as *koralon*, for rays, changing the fishery into a drift gill net fishery (Fig 7). The nets stretch from about 600 to up to 2,000m long and are 45-80m wide/deep with a mesh size of 0.61m. In 1990, the mesh size of the nets was increased to 1.83 m wide. Then sometime around 2002–2004, the mesh size reverted back to its original size. The modification of the mesh size of the net was an experiment to determine which mesh size was more efficient in catching rays. When the fishers found that fewer mobulids were caught with the larger mesh size, they reverted back to the original size. Two or three gaff hooks for pulling in the catch may or may not be used. The fishing nets used in Pamilacan varied slightly, with lengths from 700 to 2,000m and widths from 35 to 60m. The mesh size was 0.35m.

With the change in technique of fishing, the timing of activity in the fishery also changed. Fishers went out in the early afternoon and traveled four to five hours to their fishing grounds where they dropped their nets in the early evening. Boats drifted for about four to five hours or until a substantial catch was obtained. The nets were raised and the catches were secured onboard and the boats made the journey back to the fishing village. Most boats arrived by five in the morning but the time of arrival varied depending on distance of their fishing ground. If a manta was caught, the boat arrived later in the morning, usually by noon.

This modified fishery required a crew of at least five men. The “operator” acts as the captain of the boat and decides where to go. He usually also acts as the boat mechanic. The rest of the crew is responsible for dropping and raising the nets and other general duties on the boat. The fishing crew required a different set of skills. The operator had to be a skilled navigator, boat mechanic and had to have good leadership skills. The rest of the crew had to be strong enough to handle the nets. The skill of spotting the rays, a good aim, strength and good swimming ability were no longer required.

The beginning and end of the ray fishing season is signaled by changes in the direction of the wind and currents, particularly the monsoons, the southwest and the northeast winds or *habagat* and *amihan*, respectively. It is believed by fishers that this also coincides with the “leaving” and “arrival” of the animals. The change in technology allowed the fishers to extend beyond the traditional fishing season (March to May). The season for fishing for mobulids in the Bohol Sea now commences in November and ends in May, but may extend until June.

**Fig 8:**
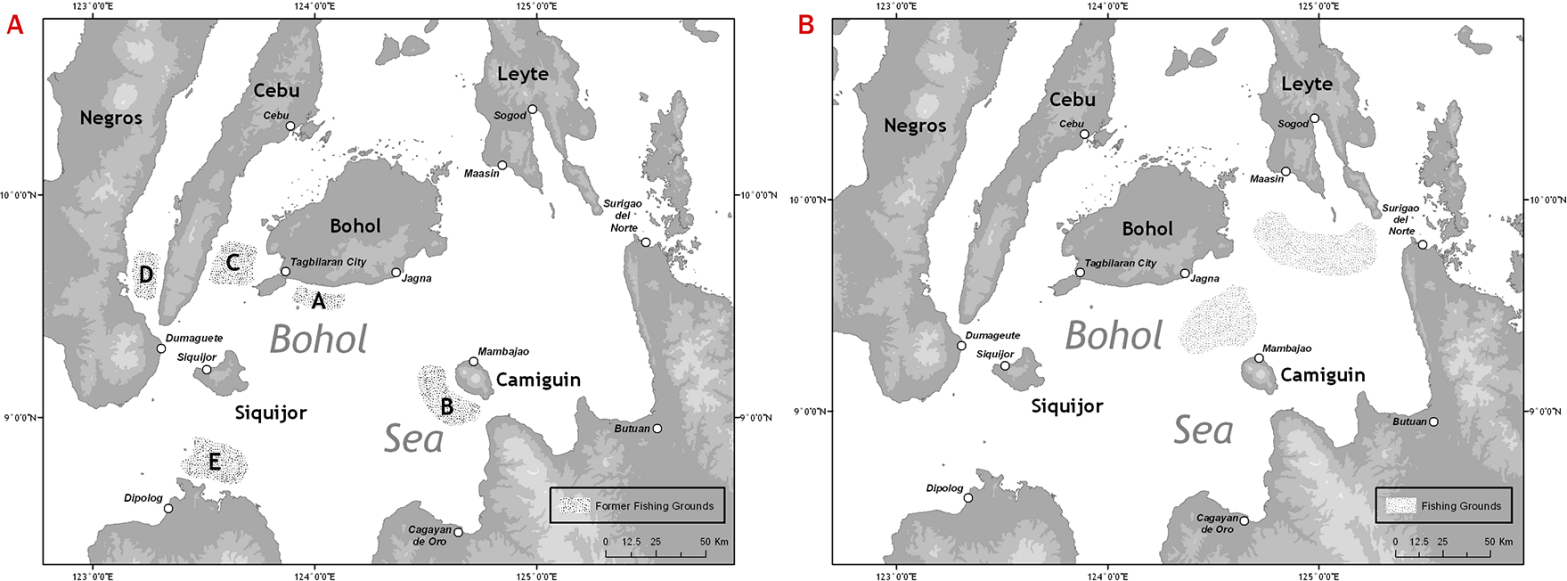
Former and current ray fishing grounds in the Bohol Sea. (A) Mobulid ray fishing grounds in 1997 according to Alava *et al*. 2002. (B) Mobulid ray fishing grounds in recent years according to Boholano fishers.

Their fishing grounds range from the North eastern part of the Bohol Sea towards Surigao del Norte, near the passage to the Surigao Strait and the southern part of the Bohol Sea close to Camiguin Island. Pamilacan fishers utilized the waters around Siquijor Island as fishing grounds as well. The fishing grounds vary depending on the direction of the currents which according to fishers change as the fishing season progresses. At the beginning of the season, fishers go South towards Camiguin, by late January or February the fishers move towards Limasawa on the northeast and by the end of the season, they will be fishing near the Surigao Strait. All fishing grounds can be reached within four to six hours from Jagna. The current ray fishing grounds are different from those identified by Alava *et al*. [34] which were concentrated at four sites (Fig 8). According to respondents this change in fishing grounds is due to declining catches, which led fishers to explore new grounds.

Mobulid fishers from Lila in Bohol, Sagay and Catarman in Camiguin continued to hunt with the use of “pumpboats” but not for as long as fishers in Jagna and Pamilacan. In Lila, ray hunting using “pumpboats” about 7 to 9m in length with single 6 to 16hp Briggs engine started in the 1970s and just like in Jagna and Pamilacan, hooks were replaced by nets. The timing of the fishery also changed and fishers used the same boats they used for whaling to catch rays with drift gill nets at night. With motorized boats fishers were able to venture to farther fishing grounds in Duero, a town in Bohol further East, Mambajao in Camiguin, Salay in Misamis Oriental and Siquijor Island in the South.

In Sagay, some fishers shifted to the use of “pumpboats” while others did not. The boats were 6-10m long with 5-16hp engines. Those that used “pumpboats” used either the same whaling harpoon or gill nets. According to respondents from Catarman, the use of motorized boats to catch manta rays began in the late 1960s. Some fishers continued hunting with non-motorized boats and harpoons until 1968 while others shifted to “pumpboats” and nets.

After mechanization of the fishery and the shift into a drift gill net fishery, the composition of the catch diversified. With nets, other species of smaller mobulids were caught. The primary product remained the same, the meat. However, in the 1970s, the market for gill rakers opened up and since then gill rakers have increased in value and now rival the market for the meat.

**Fig 9:**
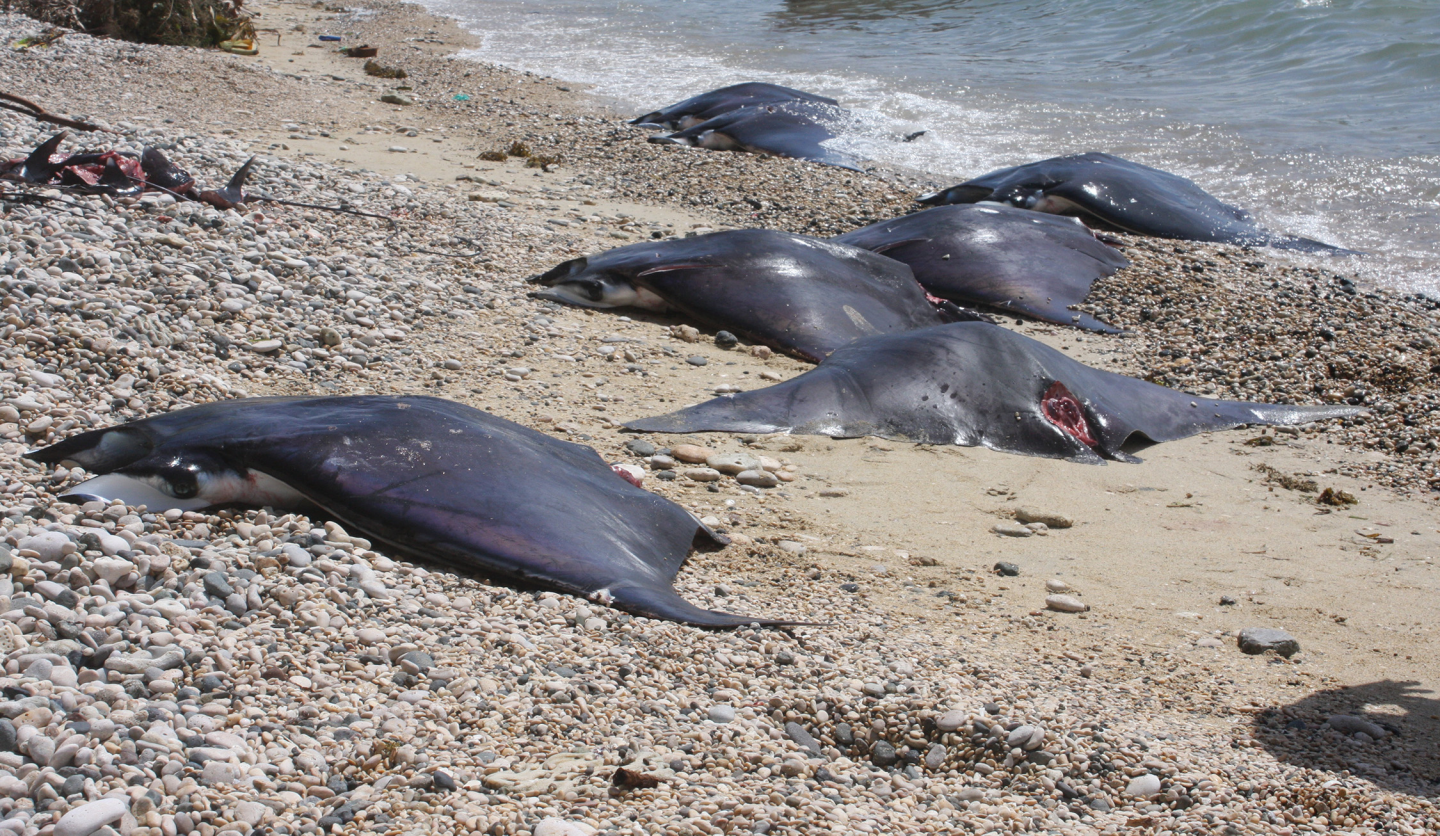
Fresh *Mobula* spp. landed on the Jagna beach.

**Fig 10:**
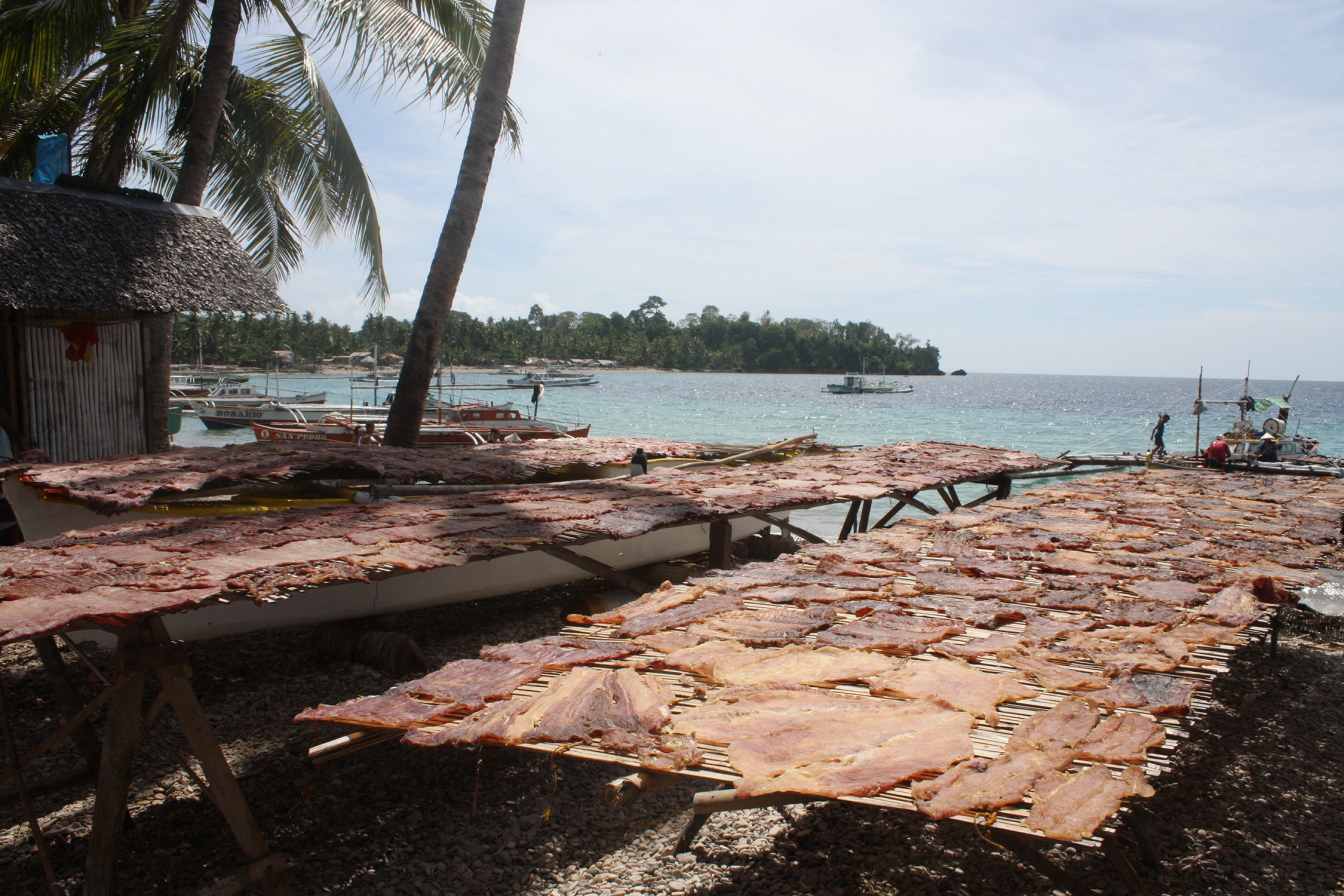
Sliced mobulid ray meat on drying racks.

As in the past, mobulids are landed and sold whole (Fig 9). However, the tail is cut off with the genital area as soon as the animal is landed and is kept by the fishers. Once purchased, each animal is cut in half from the head down to the abdomen and the internal organs are removed. Some are cut by removing the pectoral fins and then the middle part is further divided into upper and lower halves, leaving the head and thorax intact. The parts are then processed separately. The wings are the meaty parts and are processed by first carefully removing the skin. Each piece is then cut into manageable blocks of about 10 inches wide and then sliced into sheets of about 1.2 cm thick. Each sheet is rolled up and set aside. The sheets are laid out flat on a drying rack made of bamboo slats to dry for a few days (Fig 10). The liver is separated from the rest of the digestive track and set aside for cooking. The digestive tract is cleaned out and cut into smaller pieces for cooking. The gill rakers are carefully dissected and are laid out on the drying racks. The head and other more cartilaginous parts are cut into smaller pieces for either distribution to labourers or sold in the market.

Since the introduction of the market economy in the 1940s and the use of motorized boats in the 1970s the traditional system of distribution of the catch has changed. The total expenses for the fishing trip are subtracted from the sales of the catch that day and the remainder is then divided in two, with one half share for the boat owner and the other half for the crew. The crew’s half is further divided equally by the number of members, usually five.

The price of a mobulid varies depending on the species, size and the colour of the gills. Price is dictated by the market, and larger catches usually means lower prices. Consecutive days of low or no catch means higher prices for the catch on the following day. This general rule, however, does not apply for manta rays for their price has remained relatively stable for the past decade. All monetary values stated from hereon are based on the annual average Philippine Peso (Php) per US Dollar exchange rate for 2011 (1US$—Php43.31). A manta ray is much more expensive than any *Mobula* spp. An average-sized manta ray, about 3 meters wide could fetch up to Php35,000 (US$808). If the gills are black it can be worth more because white gills are cheaper. The price of a *Mobula* can vary depending on its size from as low as Php600 (US$13.85) for an animal a meter wide to Php2,000 (US$46.18) for one that is 1.5 meters wide. The colour of its gills will also affect its price. Similar to the manta ray, dark-coloured gills fetch a higher price than white-coloured gills.

**Fig 11:**
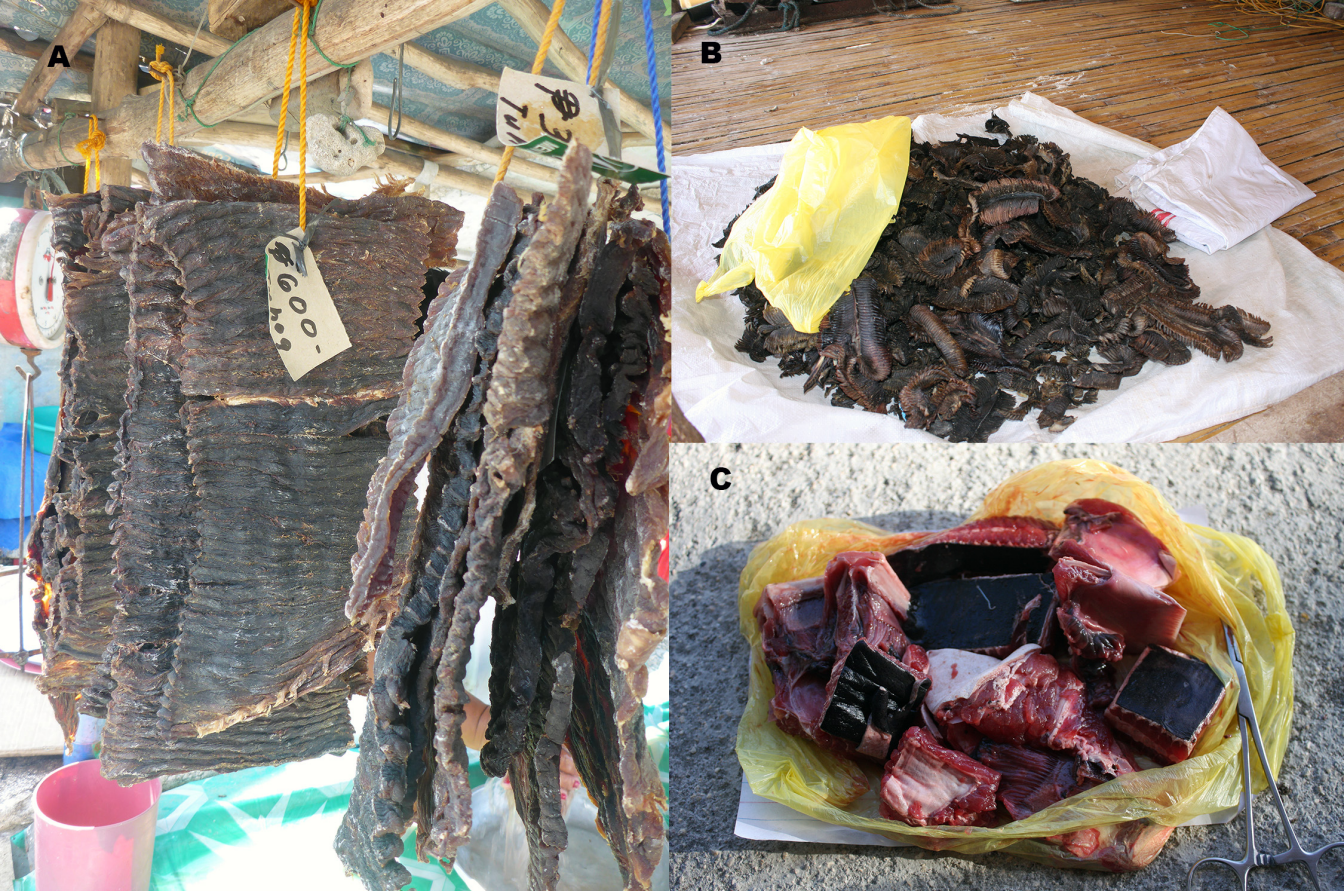
**Mobulid meat and other by-products,** (A) Dried mobulid ray meat in Jagna. (B) Dried gill rakers in Pamilacan Island. (C) Fresh mobula ray meat and head parts from the Jagna market.

Mobula meat is sold fresh and dried (Fig 11). The market (retail) price of fresh meat ranges from Php 50 to Php 60 (US$ 1.15–1.38) per kilo. The price varies depending on the part, with the fleshy part being more expensive and the smaller cartilaginous part (head part) the least expensive. Dried meat is categorized as dark and white with white meat being more expensive. However sometimes dark and white mobula meat are mixed together. Dried mobula skin is also sold per kilo. Manta ray meat is usually only sold in the market dried but there was one occasion during the study period in April 2011when fresh manta meat was sold in the Jagna market. Dried manta meat prices per kilo ranges between Php700 to Php800 (US$16.16–18.47) for dark meat and Php800 to Php1,000 (US$18.47–23.09) for white meat. Dried gill rakers are also categorized as dark and white and in contrast to dried meat, dark gill rakers are more expensive. The detailed breakdown of prices of manta and mobula meat and by-products are shown in Table 1.

**Table 1:** Prices of manta ray and *Mobula spp*. in Jagna market^a^.

	Manta ray price per kg. in Php (US$)^b^	*Mobula* spp. price per kg. in Php (US$)^b^
**Fresh meat**	90–100 (2.08–2.31)	50–60 (1.15–1.38)
**Head parts & smaller pieces**	-	70–80 (1.62–1.84)
**Dried dark meat**	700–800 (16.16–18.47)	500 (11.54)
**Dried white meat**	800–1,000 (18.47–23.09)	600 (13.84)
**Dried dark gills**	5.000 (115.44)	2,500 (57.72)
**Dried white gills**	3,000 (69.27)	-
**Dried skin**	300 (6.93)	150 (3.46)

^**a**^ Source: market survey conducted on April, November 2010; April, May 2011; December 2012; and interviews in December 2012.

^b^ The annual average exchange rate for 2011 was used: 1US$ = Php43.31.

The meat and skin of manta and mobula are primarily sold locally, within Jagna, adjacent towns and around the Bohol Province. Buyers are local, from within Jagna and adjacent towns and Pamilacan Island. The dried gill rakers, however, are sold to a middleman from Cebu who then exports them to buyers in China. Respondents were not forthcoming with details regarding the trade for gill rakers. Respondents who were buyers and boat owners did not seem to know exactly where the gill rakers were sold or exported, or what they were used for. All respondents stated that local buyers sell them to middlemen either in Cebu or Tagbilaran city who then sell them onward to China or Manila, respectively. They also stated that they think gill rakers are used by the Chinese as medicine for particular kinds of ailments. According to an investigative study, there are several anecdotes and literature that claim or suggest that gill rakers can treat ailments ranging from skin issues, throat, kidney, to fertility problems [6]. Although gill rakers are not historically considered as traditional Chinese medicine, some use it because it is believed to enhance the immune system, reduce toxins in the body, lower fever and enhance circulation of the blood [6, 35].

The change in fishing technique and mechanization of boats allowed fishers to venture to more distant fishing grounds, to stay out longer and to catch more. However, the higher capital outlay required to enter and remain in the mobulid fishery compounded by declining catch rates may have led to a decrease in the number of fishery sites as well as in the number of boats in the study sites.

In 1997, there were 22 active mobulid ray fishery sites in the Bohol Sea [34]. In 1998, after the imposition of the ban on hunting for whale sharks and manta rays in the Philippines, only six known active mobulid ray fishery sites remained, namely Pamilacan, Jagna, Hibusong, Plaridel, Lopez-Jaena, and Dipolog, two of which were new [10]. In Alava *et al*.’s [34] study, Pamilacan was identified as a secondary manta fishery site while Jagna was a newly identified site that was not confirmed nor monitored during their study.

In Lila, Bohol, the mobulid fishery ended at the same time as the whale fishery in the late 1980s. In Sagay, Camiguin by 1993, there were no more than nine pumpboats engaged in the fishery for whales (and manta rays) [36]. Similar to Lila, the fishery ended at the same time as whaling, in 1997. According to respondents from Catarman in Camiguin, by the 1980s all ray fishing boats used nets copied from Boholano and Surigao fishers who fished in Camiguin waters. The fishery ended when a local ban was imposed in the late 1990s.

In Pamilacan in 1993, there were 18 fishing boats hunting mobulids. By 1997, there were 40 boats [34]. In 2011, it was down to 14 boats. In Limasawa, it could not be determined how many fishing boats were in operation in the 1980s but according to respondents by 1984, after a storm surge brought by a typhoon hit the fishing village and destroyed the boats, the fishery ended.

In Jagna in the 1990s, there were at least 20 fishing boats targeting mobulids in two villages (Can-upao and Bunga Mar) but by 2000, some boats had been either destroyed, sold or become inactive due to bankruptcy. By 2010, there were 16 fishing boats that targeted mobulids. Out of 324 registered fishermen in the village of Bunga Mar, 115 were engaged in ray fishing. In 2011, there were 17 fishing boats targeting rays in Bunga Mar but by December 2012, there were only 15 boats in operation.

A fishing trip lasts for 15–20 hours. Boats leave between 1 and 3 pm and returned between 5 and 8am depending on where their fishing destination is located. At the beginning of the season only a few boats go out to “to test” if the rays have “arrived”. Once these boats return with a good catch, other boats follow suit. When the season is in full swing, boats go out every day, whenever the weather is favourable. The only exceptions are days just prior to a fiesta in the adjacent villages and towns, and church feast days, or holy days of obligations, recognized by the Catholic Church (i.e. Christmas, New Year, Easter, Good Friday, Feast of the Immaculate Conception).

Table 2 shows the number of catches per month during the months of monitoring in 2010 and 2011. On 2010, for a period of 16 days, from 12 April to 6 May a total of 296 mobula rays and 13 manta rays were landed. For a period of 12 days from Dec 2010 to Jan 2011, a total of 84 mobula rays and 1 manta ray were landed. On 10–11 Feb 2011, eight mantas and seven mobulas were landed. In the months of April and May 2010, an average of five boats went out in a day while in the months of December 2010 and January 2011, an average of seven boats went out in a day. For the whole month of March 2011, ten mantas were landed and an unknown number of mobula rays. For three consecutive days in April (27–29) 2011, no mantas and 12 mobula rays were landed.

**Table 2:** Number of manta and mobula rays landed at Bunga Mar in 2010 and 2011^a^.

	2010			2011				
Species	Apr (8 days)	May (8 days)	Dec (8 days)	Jan (6 days)	Feb (2 days)	Mar (31 days)	Apr (5 days)	Dec (3 days)
*Manta birostris*	5	8	0	1	8	10	2	6
*Mobula spp*.	74	223	53	31	7	No data	18	22

^a^ Source: field work from April to December 2010 and January to December 2011.

To adapt to the new fishing technique and larger market, the organization of the fishery also changed. More people were involved in the processing and marketing of the catch. The mainstay is the boat owner, who as in the earlier period plays a key role in the community being the provider of jobs and income for other members. He may no longer be the hookman or harpooner but as the owner, he provided the capital to build the boat, purchased the engine, nets and all the paraphernalia required. To engage in this fishery, the boat owner must also have had sufficient capital to finance the fishing trip which not only included covering the costs for fuel but also the expenses of the crew (i.e. food). The *labasera* or the fresh fish buyer [37] plays a central role in the marketing of mobulid products. He/she is not simply a buyer of the catch but is often also the boat owner or the wife of the boat owner. This means that he/she not only gets a share of the sales of the catch but can have the monopoly of the buying and selling of the catch, processed or not. *Labaseras* who do not own their own boat or are not directly related to a boat owner (i.e. by marriage) will sometimes finance the fishing trip. Sometimes two or more *labaseras* will pool money to finance a trip.

Canvassers are usually young able men. They swim or paddle towards the boats and “claim” a ray for a buyer. He is paid for every animal he selects. The price varies depending on the size of the animal. He carries the animal to the beach to the prospective buyer. Once sold, one or two labourers are designated to cut up the animal and carry it to the buyer’s designated spot or vehicle. The canvasser may or may not be a labourer as well. He is paid per animal cut up and delivered. The large pieces of the ray are then handled by processors, who are mostly women. They are the ones who slice the meat into thin sheets and dry them. They are paid per animal they carve up. Once processed the products (fresh or dried) are bought by market vendors or retailers. Vendors may either rent a stall at the local market or have a makeshift stall in front of their house by the side of the road. Lastly, there are the middlemen who buy fresh whole animals directly from the *labaseras* and either take it back to their home town or village to process and sell or take it to other towns to sell fresh. Gill rakers are bought by these middlemen either processed (dried) or intact with the fresh whole animal.

With the increased capital investment needed in the modernized fishery, it became necessary to form cooperations among buyers and boat owners. Money lenders emerged introducing a credit system that did not use to exist in these communities. For some communities, there was an increased dependency on fishing as their source of income.

**Fig 12:**
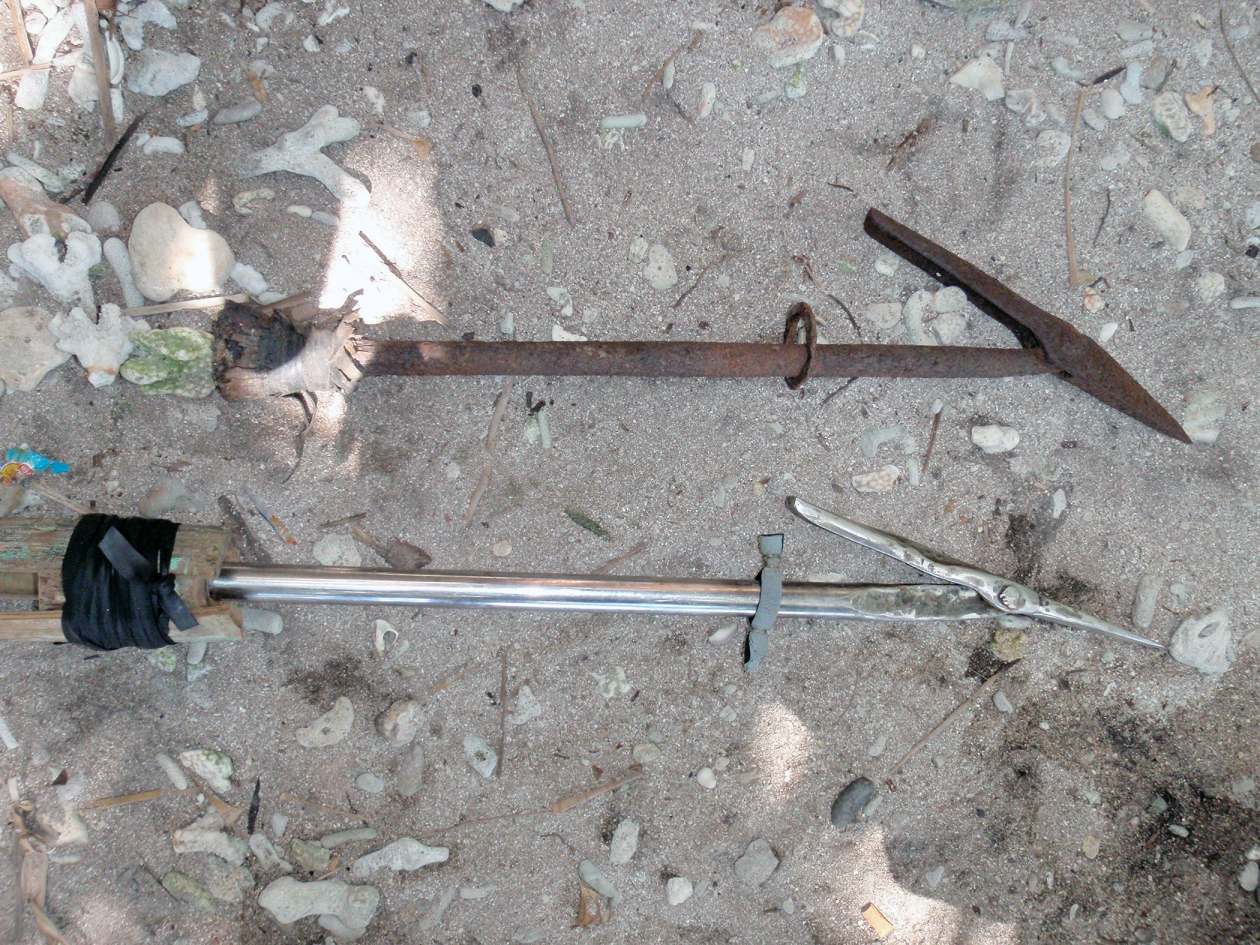
The harpoon or *untog* used to catch manta rays in Dinagat (bottom) in comparison with the *isi* (top) from Jagna.

According to respondents from Bohol, the most recent targeted mobulid fishery opened in 1998 in a village off Dinagat Island in northern Mindanao but it only became fully active in 2002. This fishery was started by a Jagna fisher. Fishers in this village use smaller motorized outrigger boats about six meters long with 16hp engines. In 2010, there were four fishing boats in the village targeting mobulids. The harpoon used was a variation from the traditional harpoon fishers on the island had used in the past (Fig 12). It was designed to specifically catch the type of manta rays that occurred in their area. According to fishers, the manta rays occur in seamounts between the islands of Homonhon, in eastern Samar and Dinagat Island. They congregate on the sandy bottom on the tops of seamounts and can be clearly seen from the surface. This allows the fishers to spot the rays easily, approach them with caution and drop a heavy harpoon on them. A crew of five to eight men on a boat set out at around 6 am. Often two boats cooperate and hunt together. They scout the area for any manta rays leaping on the surface of the water, or most often they will only set out after a report has been received the previous day from the scouting boat that rays are in the area. When they reach the seamount, a fisher will lie on the outrigger of the boat peering down into the water using goggles. Once a ray is spotted the boat approaches it slowly. While the men hold the harpoon steady, the harpooner aims the harpoon towards the target and signals the drop. The weight of the harpoon alone buries the tip in the ray’s back and the toggle secures it when the ray tries to swim away. The harpooner tries to aim at the animal’s mid-back and slightly to the side in order to kill it within seconds. Apparently if hit at the back of the head the ray dies too slowly while if hit at or near the base of the tail it will be difficult to subdue because it turns and swims upwards. This causes the rope to get tangled. It is not easy to secure a manta ray with just one hit. Quite often the fishers need to harpoon it 4 or 5 times before killing and securing it.

The harpoon tip is 63.5 cm long. It has a toggle designed similarly to that of the harpoon used in Jagna (Fig 12). The harpoon fits into a heavy metal handle about 3.66 m long, made from a steel pipe filled partly with cement. Attached to the side of the harpoon tip is a thick nylon rope about six meters long. This can be extended if needed and is secured on the boat. Once the harpoon is fastened to the manta ray, the rope is given out to let the animal tire itself out in an effort to escape. When the animal has weakened the harpooner dives in the water with a big knife to stab and kill it. He holds on to the animal’s head to keep it steady and stabs it. As soon as he has done this he surfaces, carrying the ray with him onto the boat. The ray is cut into at least three pieces and secured on the boat.

All catches are processed in the village in the same way as in Bohol. Once dried, the meat and gill rakers are transported to Pamilacan or Jagna on a bigger outrigger boat and sold to buyers there. There is no market for it in the village, or in Dinagat and Surigao. However, since the manta ray caught in this fishery is considered of inferior quality compared to that in Bohol, it fetches a much lower price. The fishing season in Dinagat begins in May and ends in October. Hence, they supply the manta ray meat in Bohol during the off-season there.

Fishers from Dipolog in Zamboanga del Norte, and Plaridel and Lopez-Jaena in Misamis Occidental are also known to catch mobulids. Although documented in 1997 by Alava *et al*. [34], these sites were not visited during this study. However, boats from Dipolog and Plaridel were documented landing their catch in Bunga Mar and in Pamilacan, respectively in 2012. Respondents from Jagna also confirmed that fishers from the aforementioned areas also fished for mobulids.

### Mobulid fishery management

There has been no formal management system for the fishery for mobulids in the Philippines. The development of fisheries laws in the Philippines was very limited until the middle of the 20^th^ century. Fishers in the 18^th^ and 19^th^ century were largely left to their own devices and marine resources were barely untouched and available for anyone to capture [38]. Even under American rule in the beginning of the 20^th^ century, there was little progress with fishery laws. The management of fisheries, particularly small-scale or subsistence fisheries, was originally the concern of the municipal governments [39]. The national government was only concerned with sectors of the fishery that produced fishery products of high commercial value such as marine mollusc fisheries, pearl fisheries and sponge fisheries [39]. From the 1950s to 1970s, a series of acts and decrees paved the way for a fisheries management system that was increasingly regulatory while still reiterating the need to develop fisheries. Although efforts were made to protect marine resources from destructive fishing, limiting or prohibiting catches of particular species, except for sponges, marine molluscs and hawksbill turtles, was still unheard of.

Traditional or local management systems were non-existent within the fishing communities that hunted mobulids. There were no traditional beliefs or taboos that limited the fishers from hunting for mobulids when they wanted, where they wanted and how much they wanted.

Concern for the fishery for large marine vertebrates only began to emerge at the end of the 1970s. However, it was in the 1990s that lobbying for fisheries reform happened and when large marine species protection laws emerged in the country. The hunting for giant manta rays was banned in the Philippines with the implementation of the Fisheries Administrative Order (FAO) No. 193 in 1998. This order was mainly aimed at prohibiting the catching, selling, purchasing, possessing, transporting and exporting of whale sharks (*Rhincodon typus*) but also included the manta rays. Several other significant pieces of legislation pertaining to fisheries followed, in particular the Philippine Fisheries Code of 1998 or the Republic Act No. 8550. Although the Fisheries Code clearly stipulated for the “protection of rare, threatened and endangered species” and the FAO No. 208 or the Conservation of rare, threatened and endangered fishery species was created in pursuant to it in 2001, it did not identify the other species of mobulids to be included in the list. Therefore, all species of *Mobula* remain unprotected. Despite the implementation of these laws, there was little evidence of enforcement particularly in Bohol. FAO 193 did not appear to have hindered the fishery in Jagna. Amidst contestations from Boholano fishers, a few months after the implementation of the ban, in 2002 there was a temporary lifting of the ban for two seasons to assess the fishery. At the end of the monitoring period, although the ban remained in place, fishers continued fishing for rays. In 2009, protests against the continuing ray fishery in the Bohol Sea resurfaced in conservation circles in the Philippines and abroad through social media networks, news and society forums [10]. In response, a rapid-resource assessment of devil rays was again conducted by the BFAR from March to May 2010 to determine if the FAO 193 was warranted [32]. This assessment was undertaken on the premise that other mobulid species were mistakenly included in the ban due to the difficulty in differentiating them from the giant manta ray [32]. Comparing the data obtained during this assessment period with that from 2002–2003 it was concluded that there was no decline in catch and that the species was not overfished [32].

Since 2010 there has been a renewed interest in protecting all species of sharks and rays in the country. Several House Bills were proposed in Congress, including House Bill 174, known as the “Sharks and Rays Conservation Act of 2010” and House Bill 5412 also known as the “Shark’s Fin Bill” filed in 2011. In 2012, two bills of similar content was also introduced, Senate Bill 2616 and House Bill No. 5880. At the time of writing, apart from readings in Congress, meetings and news media releases there has been no update on the status of all these proposed bills. To this day, there are no efforts in the local government level to regulate the mobulid fisheries. This is despite the recent clamor for protection of all elasmobranch species in the country.

Today, in Bohol, fishers claim that giant manta rays are no longer their target species but instead they target the mobula rays. Although this may be true, a giant manta ray caught is not discarded nor reported to authorities but is processed and sold. A giant manta ray has a much higher value compared to the mobula ray. In Bohol, unlike in other mobulid fisheries where the meat is not highly valued for consumption [1, 20], mobulid meat is placed high in the ranks of valued fish species. Giant manta ray meat in particular is the most expensive fish product in the local market. Compared to Sri Lanka where fishers for mobulid rays are mostly multi-day vessels [20], fishers in the Bohol Sea do not go on extended fishing trips and they land their catch immediately upon arrival on their respective fishing villages. The catch is immediately sold and processed. Neither fishing boats nor fishing villages have ice holding facilities. Fishers in Bohol are limited to catching a maximum of four giant manta rays per boat per trip because of the size of their boat. However, a catch of only one giant manta ray per boat is more common. Furthermore, unlike in Bohol, both manta and mobula rays are major by-catch in the gill net fishery in Sri Lanka [20]. Similarly, in Kerala, southern India, mobulids are caught as by-catch in the tuna gillnet fisheries using multi-day vessels [40]. The nets used have a larger mesh size ranging from 0.08–0.15m. Both meat and gill rakers are processed and sold separately. Like in Sri Lanka, the meat has a low market value and is sold either fresh or salted locally while the gill rakers fetch higher prices and are exported [40].

The socio-economic drivers of the mobulid fishery in the Bohol Sea have been at work for over a century. Although these drivers were similar across the communities studied, they were stronger in certain communities than in others, making enforcement of fishery policies and management more complicated. The hunting of manta rays has been prohibited for over 15 years but some fishers of the Bohol Sea persist in hunting them. The importance of the fishery for providing food and income to the fishing communities is evident, which makes it a major determinant of social structure [41]. In Jagna and Pamilacan, fishing is the primary source of livelihood of the people and the mobulid ray fishery is the highest income earner compared to other fisheries (i.e. flying fish, scombrids, squid). Although there is some income earned from minor sources of livelihood by other members of the family or fishers from other types of fishing, it is nothing compared to what they can earn from the ray fishery. The key actors of the fishery are the fishers, boat owners, buyers, middlemen, sellers, market vendors or retailers, labourers and processors, all of whom benefit from the fishery. In addition, other members of the fishing community, as well as adjacent villages, benefit directly or indirectly from the fishery.

In Jagna, where hunting of mobulids has been practiced for over a century, this way of life has become embedded in their culture, making ties to it ever stronger. All respondents interviewed in the village openly admitted that their village specializes in mobulid ray fishing and that the fishery has been practiced “since olden times”. Fishers expressed a sense of self and identity when they repeatedly emphasized that “fishing is their way of life”. Another way to understand their response is that being a hunter of large marine vertebrates is a true demonstration of their manhood or masculinity. The harpooners and hookmen of the Bohol Sea were the “heroes” of their day. It was a privilege and honour that was not inherited but rather earned. The risk involved in their task, and the dependence of the success of the fishery on their skills, made the harpooner and hookman one of the most prominent individuals in their communities. This notability has not faded to this day. The biggest trader and boat owner on Pamilacan Island was one of the most successful hookmen on the island. Although he no longer joins the fishing trips, he is still looked upon as the fishing expert on the island. Similarly in Jagna, one of the most prominent harpooners is also the owner of one of the most successful fishing boats in the village. He is still respected in all matters pertaining to the ray fishery.

Although the role of the harpooner and hookman has disappeared with the change in the fishing technology, belonging to a ray fishing boat group still has a certain prominence attached to it. It may not involve the same kind of skill and courage, but the mere difficulty of life at sea and the risk of engaging in an unpredictable endeavor amidst the hazards of the sea brings its own merit and rewards. These attributes of bravery and masculinity attributed to fishers for their ability to catch “big fish” and overcome dangerous difficulties at sea are not unique and have been also described in other types of fisheries in other places in the Philippines [42, 43,44]. As Fabinyi [42] notes, however, fishing remains a low-paying and difficult job despite the “status, pride and satisfaction” ascribed to it. However, with the ray fishery, there is a possibility of “making it big” or earning a windfall income depending on one’s perseverance and “luck”. It is this possibility that continues to lure newcomers into the fishery and encourages “old timers” to stay in the industry.

This study attempted to reconstruct the marine environmental history of the Bohol Sea by investigating its mobulid fishery. In fisheries studies such as this where data are scarce it is even more important to make use of non-traditional historical data to set baselines for marine animals and ecosystems [45]. Similarly, it was important to examine the history of the people’s interactions with their marine environment by examining the technical side of the fisheries. Given the dearth of available literature and data on this type of fishery, it was necessary to utilize a combination of unpublished and published documentary materials and the ethnographic evidence from oral history.

## Conclusions

The fisheries for mobulid rays have been practiced for over a century in the Bohol Sea. The fishery has evolved and methods have changed. The size of the fishing fleet targeting mobulid rays in the Bohol Sea has decreased since the 1920s but the extent of the fishing grounds has changed and expanded, towards the Surigao Strait and fishing efficiency increased. The market has expanded from purely local to other islands and provinces within the country. The market for gill rakers, in particular, has expanded to international markets, mainly China. The monetary value of its by-products has increased making it one of the most expensive marine fish products in the region.

Four species of mobulids have been verified to be caught in the Bohol Sea: *Manta birostris*, *Mobula japanica*, *Mobula thurstoni* and *Mobula tarapacana*. A fifth species, targeted off Dinagat, is most likely the *Manta alfredi*. Although *Mobula eregoodootenke* and *Mobula kuhlii* were reported to be caught in Bohol [32], none were identified in this study.

Based on available data the fishery for mobulids has been practiced the longest in Jagna. Currently, Jagna is the center of the mobulid ray fishery in the Bohol Sea. It is where other mobulid ray fisheries in the country converge and radiate from. Pamilacan also plays a significant part in the fishery. Bohol and Dinagat may be the only two provinces with a targeted fishery for mobulid rays in the country. In other areas in the Visayas, mobulids are caught as by-catch.

There are still gaps in the knowledge on the extent of the mobulid ray fishery in the Bohol Sea. Other fishery sites in Camiguin, Southern Leyte, Cebu, northwestern Mindanao need to be investigated to determine whether mobulids are targeted or by-catch. Reports of mobulids being caught and sold in other regions in the Philippines still persist and should be investigated for a more comprehensive understanding of the mobulid fisheries.

Almost two decades after the implementation of the ban on the fishing and trade of giant manta ray, there is little evidence of enforcement of the law. All other species of mobulids including the reef manta ray remain unprotected. Despite the introduction of other fishery management regulations, weak enforcement means the fishery for mobulids in the Philippines is still effectively an open access one. The characteristics of the mobulid fishery in the Bohol Sea are unique compared to other reported mobulid fisheries in the world active today. Mobulids are the target species of fishing boats using drift gill nets, and all boats go on one-day trips. The meat is the primary product of the fishery while the gill rakers became a recent additional product. The giant manta ray fetches the highest price in the market for both meat and gill rakers. This fishery merits a thorough assessment of the fishing effort, market and trade as well as the main drivers of the fishery in order to design a more appropriate management strategy.
